# Impaired neutralisation of SARS-CoV-2 delta variant in vaccinated patients with B cell chronic lymphocytic leukaemia

**DOI:** 10.1186/s13045-021-01219-7

**Published:** 2022-01-09

**Authors:** Helen Parry, Graham McIlroy, Rachel Bruton, Sarah Damery, Grace Tyson, Nicola Logan, Chris Davis, Brian Willett, Jianmin Zuo, Myah Ali, Manjit Kaur, Christine Stephens, Dawn Brant, Ashley Otter, Tina McSkeane, Hayley Rolfe, Sian Faustini, Alex Richter, Sophie Lee, Farooq Wandroo, Salim Shafeek, Guy Pratt, Shankara Paneesha, Paul Moss

**Affiliations:** 1 Institute of Immunology and Immunotherapy, University of Birmingham, Birmingham, B15 2TT UK; 2 Institute of Cancer and Genomic Sciences, University of Birmingham, Birmingham, B15 2TT UK; 3 Institute of Applied Health Research, University of Birmingham, Birmingham, B15 2TT UK; 4 MRC- University of Glasgow Centre for Virus Research, University of Glasgow, Glasgow, G61 1QH UK; 5 UK Health Security Agency, Porton Down, Salisbury, SP4 OJG UK; 6 Cancer Research UK Clinical Trials Unit, University of Birmingham, Birmingham, B15 2TT UK; 7 Department of Haematology, The Royal Wolverhampton NHS Trust. Wolverhampton Hospital, Wolverhampton, WV10 0QP UK; 8 Department of Haematology. Sandwell and West Birmingham, NHS Trust, Birmingham, B18 7QH UK; 9 Department of Haematology, Worcestershire Acute Hospitals NHS Trust, Worcester, WR5 1DD UK; 10 Queen Elizabeth Hospital, University Hospitals Birmingham, Birmingham, B15 2TH UK; 11 Birmingham Heartlands Hospital, University Hospitals Birmingham, Birmingham, B9 5SS UK

**Keywords:** Vaccination, CLL, Leukaemia, SARS-CoV-2, COVID, Antibody

## Abstract

**Background:**

Immune suppression is a clinical feature of chronic lymphocytic leukaemia (CLL), and patients show increased vulnerability to SARS-CoV-2 infection and suboptimal antibody responses.

**Method:**

We studied antibody responses in 500 patients following dual COVID-19 vaccination to assess the magnitude, correlates of response, stability and functional activity of the spike-specific antibody response with two different vaccine platforms.

**Results:**

Spike-specific seroconversion post-vaccine was seen in 67% of patients compared to 100% of age-matched controls. Amongst responders, titres were 3.7 times lower than the control group. Antibody responses showed a 33% fall over the next 4 months. The use of an mRNA (*n* = 204) or adenovirus-based (*n* = 296) vaccine platform did not impact on antibody response. Male gender, BTKi therapy, prophylactic antibiotics use and low serum IgA/IgM were predictive of failure to respond. Antibody responses after CD20-targeted immunotherapy recovered 12 months post treatment. Post-vaccine sera from CLL patients with Spike-specific antibody response showed markedly reduced neutralisation of the SARS-CoV-2 delta variant compared to healthy controls. Patients with previous natural SARS-CoV-2 infection showed equivalent antibody levels and function as healthy donors after vaccination.

**Conclusions:**

These findings demonstrate impaired antibody responses following dual COVID-19 vaccination in patients with CLL and further define patient risk groups. Furthermore, humoural protection against the globally dominant delta variant is markedly impaired in CLL patients and indicates the need for further optimisation of immune protection in this patient cohort.

**Supplementary Information:**

The online version contains supplementary material available at 10.1186/s13045-021-01219-7.

## Introduction

Patients with CLL suffer from relative immune suppression due to the underlying disease and the impact of CLL-directed therapy [1]. Infection remains a significant cause of morbidity and mortality and SARS-CoV-2 infection is associated with poor clinical outcome [2, 3]. Mortality rates appear to have fallen somewhat between the first and second waves of the pandemic, potentially reflecting improvements in clinical care, but remain high [4]. The introduction of highly efficacious COVID-19 vaccines has transformed global control of the pandemic but the optimisation of vaccine delivery for patients with CLL remains uncertain.

Patients with CLL develop a suboptimal humoural response following dual COVID-19 vaccination with responses seen in only 40–60% [5–7]. These values vary according to disease stage but are particularly suppressed in those on active therapy [6]. As such it is important that accurate determinants of antibody response are elicited in large patient cohorts in order to allow optimal design of vaccine delivery. A number of questions remain unresolved including the potential impact of vaccine subtype and the temporal kinetics of vaccine response recovery following previous treatment.

A further challenge is the emergence of the SARS-CoV-2 delta variant and its potential impact on the efficacy of vaccine responses [8]. Several SARS-CoV-2 variants of concern have arisen during the COVID-19 pandemic and differ in a range of biological properties including efficiency of transmission and relative evasion of natural or vaccine-induced SARS-CoV-2-specific immune responses [9]. The delta variant is now globally dominant and more than twice as contagious as the Wuhan virus [10]. Importantly, current COVID-19 vaccines contain the spike protein sequence from the original Wuhan virus whereas the delta variant contains four further mutations [11]. Post-vaccine sera from healthy donors show relative loss of neutralising activity against the delta variant [12] but it is unknown how vaccine-induced antibody responses compare for patients with CLL. This is particularly important given lower spike-specific antibody responses in patients and suboptimal functional responses against other pathogens [13].

Following our interim report on 50 dual vaccinated patients within the CLL-VR study [4], we have now analysed vaccine-induced serological responses in a large cohort of 500 patients with CLL following two different vaccine regimens. We define additional patient subgroups at risk for negative response and for the first time, demonstrate marked impairment of neutralisation of the SARS-CoV-2 delta variant.

## Methods

### Study design and participants

Patients with a confirmed diagnosis of CLL or small lymphocytic leukaemia (SLL) were recruited with no additional exclusion criteria. Informed consent was obtained by remote consultation and work performed under the CIA UPH IRAS approval (REC 20\NW\0240) from North-West and Preston ethics committee and conducted according to the Declaration of Helsinki. The dates and type of SARS-CoV-2 vaccination were obtained with self-reported information on stage and date of CLL diagnosis, CLL treatment and infection history as previously described [5]. Participant demographics can be found in Table 1.

**Table 1:** Participant demographics

	Whole cohort	Neutralisation assay cohort
Number of patients	500	94
*Age (years)*		
Median	67	71
IQR	60 to 72	64 to 77
Range	39 to 89	50 to 89
*Sex*		
Men	267 (53%)	53 (56%)
Women	233 (47%)	41 (44%)
*Vaccine received*		
Pfizer	204 (41%)	48 (51%)
AstraZeneca	296 (59%)	46 (49%)
*Vaccine interval*		
3-weeks	16 (3%)	10 (11%)
Delayed	484 (97%)	84 (89%)
*Delayed vaccine interval (days)*		
Median	77	77
IQR	70 to 79	74 to 80
Range	33 to 133	43 to 112
*Time from second vaccine to blood test (days)*		
Median	20	28
IQR	17 to 29	20 to 37
Range	4 to 133	10 to 66
*Time since CLL diagnosis (months)*		
Median	73	88
IQR	34 to 133	37 to 162
Range	1 to 408	5 to 276
*CLL stage at diagnosis*		
A	429 (86%)	81 (86%)
B	30 (6%)	4 (4%)
C	41 (8%)	9 (10%)
*Previous treatment*		
Watch and wait	279 (56%)	57 (61%)
Treatment planned	13 (3%)	1 (1%)
1 line	128 (26%)	25 (27%)
2 lines	48 (10%)	7 (7%)
3 + lines	32 (6%)	4 (4%)
On BTKi	99 (20%)	20 (21%)
On venetoclax	21 (4%)	1 (1%)
Previous chemotherapy	143 (29%)	24 (26%)
Previous anti-CD20	153 (31%)	24 (26%)
History of infection Frequent infections	145 (29%)	23 (25%)
Hospitalisation with infection	95 (19%)	14 (15%)
Prophylactic antibiotics	37 (7%)	8 (9%)
IVIG	41 (8%)	2 (2%)
Immunoglobulin deficiency Number	471	94
IgG (< 6 g/L)	236 (50%)	40 (43%)
IgA (< 0.8 g/L)	232 (49%)	38 (40%)
IgM (< 0.5 g/L)	177 (38%)	46 (49%)

Samples were obtained 2–3 weeks following the second vaccination and again up to 30 weeks later (median 16 weeks; range 10–30). Local participants undertook phlebotomy whilst those distant, donated a dried blood spot sample (DBS). A total of 93 healthy donor controls were recruited from local primary care networks.

## Procedures

### Roche Elecsys® electrochemiluminescence immunoassay (ECLIA)

Using ECLIA, qualitative IgG/A/M Anti-nucleocapsid protein (NP) antibodies specific to SARS-CoV-2 were detected (COV2, Product code: 09203079190); cut-off index value ≥ 1.0 considered positive for anti-nucleocapsid antibodies. Using the quantitative ECLIA assay, anti-spike (S) receptor binding domain antibodies were detected (COV2 S, Product code 09289275190) with values ≥ 0.8 U/ml considered positive. DBS eluates required multiplication by factor 13.6 to account for dilution factor following validation (*r* = 0.98; *p* < 0.0001) (Additional file 1: figure S1).

### Dried blood spot ELISA analysis

Dried blood spot (DBS) analysis was carried out as previously described [14]. IgG, IgA and IgM antibody isotypes against stabilised trimeric SARS-CoV-2 spike glycoprotein are reported with a positive result classed as a ratio of 1 or more.

### Serum Immunoglobulin concentration

Quantification of IgG, IgA and IgM was evaluated using the COBAS 6000 (Roche) at the University of Birmingham Clinical Immunology Service as previously described [4].

### Neutralisation and pseudoneutralisation assay

A549-ACE2-TMPRSS2 cells [15] were seeded at a cell density of 1 × 10^4^/well in 96-well plates 24 h before inoculation. Serum was titrated starting at a 1:100 dilution. Virus was incubated at a multiplicity of infection (MOI) of 0.01 with serum for 1 h prior to infection. All wells were performed in triplicate and at 72 h after infection plates were fixed with 8% formaldehyde and stained with Coomassie blue for 30 min. Plates were washed and dried overnight before quantification using a Celigo Imaging Cytometer (Nexcelom) to measure the staining intensity. Percentage cell survival was determined by comparing the intensity of the staining to uninfected wells. Maximal virus neutralisation was defined as relative improvement in cell survival following addition of 1:100 serum dilution.

### Pseudotyped-virus neutralisation

HEK293, HEK293T and 293-ACE2 cells were maintained in Dulbecco’s modified Eagle’s medium (DMEM) supplemented with 10% foetal bovine serum, 200 mM L-glutamine, 100 µg/ml streptomycin and 100 IU/ml penicillin. HEK293T cells were transfected with the appropriate SARS-CoV-2 spike gene expression vector in conjunction with lentiviral vectors p8.91 and pCSFLW using polyethylenimine (PEI, Polysciences, Warrington, USA). HIV (SARS-CoV-2) pseudotype-containing supernatants were harvested 48 h post-transfection, aliquoted and frozen at − 80 °C prior to use. The SARS-CoV-2 spike glycoprotein expression constructs for Wuhan-Hu-1, B.1.617.2 have been described previously [16]. The delta construct bore the following mutations relative to the Wuhan-Hu-1 sequence (GenBank: MN908947): T19R, G142D, E156del, F157del, R158G, L452R, T478K, D614G, P681R, D950N. 293-ACE2 target cells were maintained in complete DMEM supplemented with 2 µg/ml puromycin.

Neutralising activity in each sample was measured by a serial dilution approach. Each sample was serially diluted in triplicate from 1:50 to 1:36,450 in complete DMEM prior to incubation with approximately 1 × 10^6^ CPS per well of HIV (SARS-CoV-2) pseudotypes, incubated for 1 h, and plated onto 239-ACE2 target cells. Luciferase activity was quantified after 48–72 h by the addition of Steadylite Plus chemiluminescence substrate and analysis on a Perkin Elmer EnSight multimode plate reader (Perkin Elmer, Beaconsfield, UK). Antibody titre was then estimated by interpolating the point at which infectivity had been reduced to 50% of the value for the ‘no serum’ control samples.

### Statistical analysis

For comparative analysis, Mann–Whitney U-tests or Spearman rank correlation were performed and data presented as geometric means. Kruskal–Wallis was performed with post-hoc Dunn’s analysis for comparative groups and Wilcoxon’s matched-pairs signed rank test for paired responses. Logistic regression of clinical variables was tested for associations with positive antibody response after second vaccine. Chi-square analysis was used to compare proportions of responders. Analysis was performed using Graphpad prism v9.1.0 for Mac (San Diego, California USA) and SPSS Statistics v27.0 for Windows (Armonk, NY: IBM Corp.)

## Results

### Patient characteristics

A total of 502 participants were recruited together with 93 age-matched healthy donor controls. Two patients were excluded from analysis as they had received prophylactic monoclonal spike-specific antibody therapy. The median age of the 500 patients was 67 years (IQR 60–72) and 53% were male. 204 (41%) had received the BNT162b2 vaccine (Pfizer/BioNTech) and 296 (59%) received the ChAdOx1 vaccine (Oxford/AstraZeneca). About 484 (97%) received the vaccine on an ‘extended interval’ (median of 11 weeks between doses) whilst 16 (3%) received the BTN162b2 vaccine on a standard 3-week interval. The median time to sample collection following the second vaccine was 20 days (IQR 17–29) (Table 1).

A total of 279 patients (56%) were Binet stage A, untreated and under expectant monitoring. 208 had received therapy, of which 128 had received one line of therapy. 99 of the 208 were currently taking a Bruton Tyrosine Kinase inhibitor (BTKi) (20%) whilst 21 were on active therapy with a BCL-2 inhibitor. A total of 13 patients (3%) were due to commence therapy imminently.

145 patients (29%) reported a clinical history of frequent infections whilst 95 (19%) also reported a previous hospital admission for infection. 37 (7%) patients were on prophylactic antibiotics for a history of recurrent infections and 41 (8%) were on immunoglobulin replacement therapy (IVIG).

### Antibody responses are seen in 67% of patients compared to 100% of age-matched controls and titres are 3.7-fold lower in those who respond

**Fig. 1:**
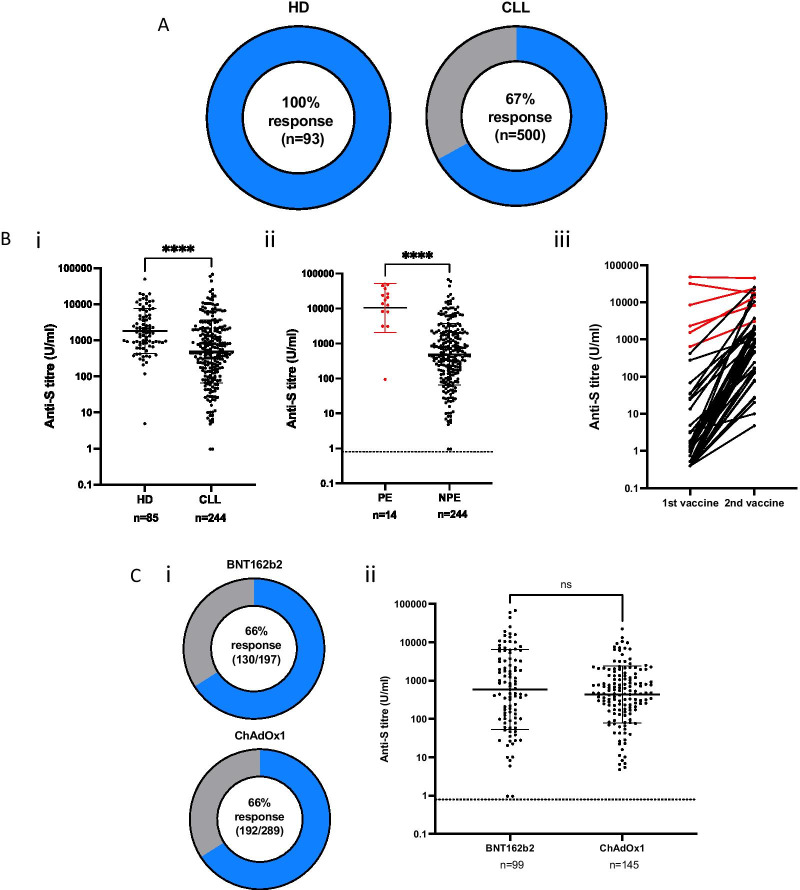
Prevalence and titre of SARS-CoV-2 spike-specific antibody response in patients with CLL following dual COVID-19 vaccination. **A** Proportion of donors who develop a positive antibody response following dual vaccination. HD = Healthy donors; CLL = CLL-VR patient cohort. **B** (i) Antibody titres in participants with a positive antibody response. Donors with serological evidence of previous natural SARS-CoV-2 infection (previous exposure; PE) were excluded (*p* =  < 0.0001). (ii) Antibody titres in CLL patients in relation to history of previous natural exposure (PE) or no previous exposure (NPE) (*p* < 0.0001). Cut off for positive response is indicated by dotted line). (iii) Antibody titres in paired serum samples from patients with CLL following first and second vaccine. Red lines indicate patients with PE. **C** (i) Proportion of patients with CLL who develop a positive antibody response following dual vaccination with either the BNT162b2 or ChAdOx1 vaccine. (ii) Antibody titres in CLL patients in relation to vaccine subtype

A serological anti-S response was identified in 67% of participants (336/500) compared to 100% (*n* = 93) of healthy controls (Fig. 1A) following ELISA analysis of eluate from DBS collection. 14 participants had serological evidence of previous SARS-CoV-2 infection (‘Previous exposure’: PE) as determined by the presence of a nucleocapsid-specific antibody response, of which 4 had been asymptomatic. This group of 14 was representative of the total cohort as 6 patients were on expectant management, 1 was on BTKi therapy, 4 were IgA deficient, 1 was on IVIG and 2 were on antibiotic prophylaxis. These 14 donors were excluded from subsequent analysis. The overall serological response rate within infection-naive patients was 66% (322/486).

Quantitative antibody titres were next measured by the ROCHE electrochemiluminescence immunoassay using serum eluates from patients who had shown a positive DBS response. Sufficient eluate was available for 258 of the 336 samples. Spike-specific antibody titres were markedly reduced in patients compared to age-matched control donors (HD; *n* = 93) (HD: geomean 2110 U/ml (SD 4.4) vs CLL:579 U/ml (SD 8.2); *p* < 0.0001). A 3.7-times reduction in titre was observed in infection-naive patients after exclusion of donors with previous natural infection (HD: 1,821 U/ml (SD 4.2) vs CLL: 490 (SD 7.5), *p* < 0.0001) (Fig. 1B (i)). Antibody titres in patients with previous infection were 21-times higher than those seen in patients without previous infection (10,470 U/ml (SD 5) vs 490 U/ml (SD 7.5) U/ml; *p* < 0.0001) Fig. 1B (ii)).

Paired samples following the first and second vaccine were available in 43 patients with a positive response to the second vaccine. A 294-fold incremental improvement was seen in patients with no previous infection (NPE) (mean 1.93 (SD 7.3) vs 568 U/ml (SD 7.9))*.* (Fig. 1B (iii). This was reduced to only 2.4-fold in 6 donors with previous infection due to markedly improved responses to the first vaccine (mean 5,620 U/ml (SD 5.6) vs 13,710 U/ml (SD 2.5)).

### Antibody responses are comparable with mRNA or adenovirus-based vaccine platforms

We next compared antibody response rates in relation to vaccine subtype amongst the 500 participants. Within infection-naive patients, a positive antibody response developed in 66% (130/197) of both BNT162b2 mRNA vaccine and ChAdOx1 adenovirus-based vaccine recipients (192/289)) (Fig. 1C (i)). Furthermore, no difference was observed in relation to antibody titre amongst patients who developed a response (BNT162b2: mean 582 (SD 11) vs ChAdOx1 436 (SD 5.5) U/ml; *p* = 0.26) (Fig. 1C (ii)).

The time interval between 1st and 2nd vaccine dose can influence antibody responses and comparison of participants with a positive antibody response who received vaccination on a 10–12 week ‘extended interval’ regimen showed higher median titres compared to those with a standard 3-week interval (633 U/ml (SD 8.0) vs 93 U/ml (SD 6.1); *p* = 0.0014) (Additional file 2: Figure S2). This differential was also observed in analysis of recipients who received the BTN162b2 vaccine (825 vs 90 U/ml *p* = 0.01) although the median age was higher in those with the 3-week interval (82 vs 67 years).

### Vaccine response rates vary across the treatment course of CLL

**Fig. 2:**
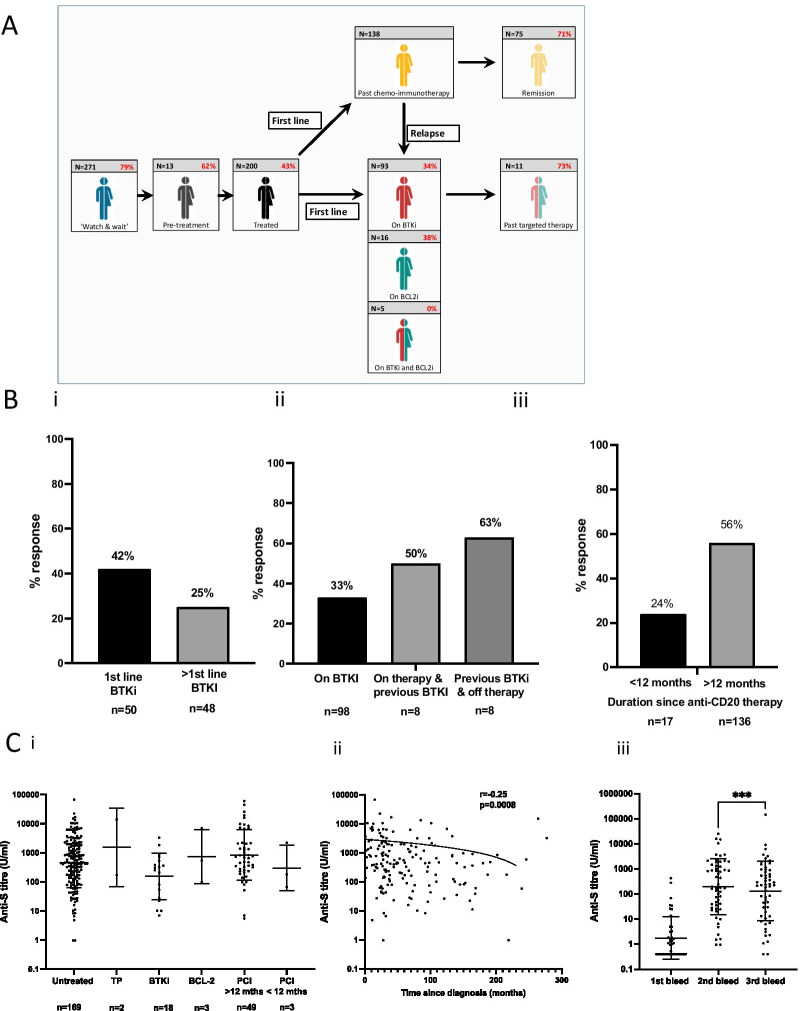
SARS-CoV-2 spike-specific antibody responses after vaccination in patients with CLL in relation to stage of disease. **A** Infographic to show the proportion of patients who develop an antibody response after COVID-19 vaccination in relation to disease stage and management (*n* = 484). **B** (i) Proportion of patients on BTKi therapy who develop a positive antibody response by line of therapy (*p* = 0.07) and (ii) in relation to current or previous BTKi therapy (p = NS). (iii) The proportion of patients with a positive antibody response who had received an anti-CD20 therapy within 12 months or longer (*p* = 0.008). **C** (i) Antibody titres in CLL patients who develop an positive antibody response (*n* = 244). Antibody titres do not vary across the disease course. (Untreated; Treatment planned (TP); Bruton Tyrosine Kinase therapy (BTKi); BCL-2 inhibitor (BCL-2); Previous Chemo-immunotherapy but not on active therapy (pCI). (ii). Antibody titres in relation to ‘time since diagnosis’ in patients who develop an antibody response and are on expectant ‘watch and wait’ management (*r* =  − 0.25; *p* = 0.0008). (iii) Antibody titres are broadly maintained at 4 months after vaccination. Serial titres at 5-weeks after first vaccine, 2–3 weeks after second vaccine and 4-months (range 3–8) after second vaccine (*n* = 56) (*p* = 0.0006 Wilcoxon paired analysis between 2nd and 3rd bleed time point)

Given the clinical heterogeneity amongst patients with CLL we next assessed antibody responses in relation to clinical status and management. Patients were divided into 5 groups across the patient journey: ‘watch and wait’; ‘plans to start treatment imminently’; ‘current treatment with BTK inhibitor’; ‘current treatment with venetoclax’ (BCL2 inhibitor) and; ‘previously completed chemo-immunotherapy’. The 14 patients with serological evidence of previous natural infection were not included in assessment and an additional two patients were not assigned due to therapy with steroids or radiotherapy alone (Fig. 2A).

Antibody response rates from dried blood spot eluates were highest amongst patients with Binet stage A disease who were on expectant ‘watch and wait’ management, where 79% showed a positive response (214/271). A total of 13 patients were enrolled in whom treatment was being actively planned. The antibody response rate in this group was somewhat lower at 62% (8/13) and likely reflects the immune suppressive impact of progressive disease.

The overall antibody response rate in patients who were on treatment or had previously received therapy was 43%. The rate in those on BTKi therapy was low at 32% (32/98) and in line with previous reports [6, 7]. These values were higher amongst patients receiving BTKi as first line therapy (42%) compared to those receiving it at relapse (25%) (*p* = 0.07) (Fig. 2B (i)). A trend toward improvement in response was also seen amongst patients who had stopped BTKi therapy where response rates were 50% or 63% depending on status of current therapy (Fig. 2B (ii)). A total of 21 patients were on treatment with a BCL2 inhibitor and here the antibody response rate was 29% (6/21). None of the 5 patients on dual BTKi and BCL2-inhibitor therapy made a vaccine response. No samples were taken from patients on active chemo-immunotherapy but 71% of patients who had previously been treated with chemo-immunotherapy and remained in remission showed a serological response.

### Antibody responses to vaccination improve in patients after 12 months since completion of anti-CD20 therapy

CD20-targeted antibody therapy has a major impact on the rate and magnitude of the antibody response to COVID-19 vaccination. However, it is unclear how long this effect lasts after completion of therapy in patients with CLL. Importantly, markedly higher rates of antibody response were seen in patients who had completed anti-CD20 therapy more than 12 months prior to sample donation compared to those who were less than 12 months since cessation of therapy (median 2 months IQR 1–12), with vaccine responses of 56% and 24%, respectively (*p* = 0.008) (Fig. 2 B (iii)).

### Antibody titres in those patients who do respond are comparable and decline by 33% at 4-months after second vaccine

We next evaluated antibody titres amongst those participants with a serological response in relation to disease stage (*n* = 244). Interestingly, titres were broadly equivalent in all subgroups (Fig. 2 C (i) (*p* = 0.09)). Titres were also assessed within the ‘watch and wait’ group in relation to ‘time since diagnosis’ and were found to decrease with increasing length of follow up (*r* =  − 0.25; *p* = 0.0008) (Fig. 2C (ii)). Serum immunoglobulin levels were measured in this group too and showed a gradual decrease during follow-up which was significant for IgM (Additional file 3: Figure S3).

In order to assess the relative stability of antibody responses, samples were also taken at a median of 4 months (range 10–32 weeks) after the second dose from 56 infection-naive participants. Titres were seen to fall by 33% from the peak value post second vaccine (mean: 197 U/ml (SD 13) vs 132 U/ml (SD 15) *p* = 0.0006 Wilcoxon) (Fig. 2 C (iii)).

### Male gender, BTKi therapy and low serum immunoglobulin levels are predictors of failure to generate an antibody response after vaccination

The importance of individual clinical and laboratory variables on the probability of developing an antibody response after second vaccination was next defined within the CLL-VR cohort (Table 2).

**Table 2:** Determinants of positive antibody response after dual COVID-19 vaccine in patients with CLL

Variable	Univariate analysis		Multivariate analysis (*n* = 459)	
	Odds ratio (95% CI)	*P* value	Odds ratio (95% CI)	*P* value
Age (increasing)	0.99 (0.96 to 1.01)	0.160	0.98 (0.96 to 1.01)	0.265
Sex (male)	0.64 (0.44 to 0.94)	0.022	0.56 (0.34 to 0.91)	0.020
Vaccine type (Pfizer)	0.98 (0.67 to 1.44)	0.919	1.16 (0.71 to 1.91)	0.553
Duration of CLL (increasing)	0.99 (0.99 to 0.99)	< 0.0001	0.99 (0.99 to 1.00)	0.334
CLL treatment (yes)	0.27 (0.19 to 0.41)	< 0.0001	0.71 (0.22 to 2.27)	0.563
BTKi treatment (yes)	0.16 (0.10 to 0.27)	< 0.0001	0.20 (0.08 to 0.48)	< 0.0001
Previous BTKi treatment (yes)	0.65 (0.24 to 1.76)	0.393		
Venetoclax treatment (yes)	0.19 (0.07 to 0.50)	0.001	0.31 (0.09 to 1.04)	0.059
Previous Venetoclax treatment (yes)	0.76 (0.13 to 4.60)	0.767		
Previous chemotherapy (yes)	0.41 (0.27 to 0.62)	< 0.0001	2.24 (0.32 to 15.64)	0.415
Previous anti-CD20 (yes)	0.43 (0.29 to 0.64)	< 0.0001	1.88 (0.52 to 6.82)	0.338
Previous immuno-chemotherapy (yes)	0.44 (0.29 to 0.66)	< 0.0001	0.46 (0.06 to 3.52)	0.453
Planned treatment (yes)	0.81 (0.26 to 2.52)	0.716		
History of recurrent infection (yes)	0.49 (0.33 to 0.74)	0.001	0.96 (0.53 to 1.73)	0.882
Prophylactic antibiotics (yes)	0.13 (0.06 to 0.29)	< 0.0001	0.28 (0.09 to 0.86)	0.027
IVIG (yes)	0.09 (0.04 to 0.20)	< 0.0001	0.34 (0.11 to 1.02)	0.054
IgA (< 0.8 g/L)	0.19 (0.12 to 0.30)	< 0.0001	0.28 (0.16 to 0.48)	< 0.0001
IgG (< 6 g/L)	0.51 (0.35 to 0.76)	0.001	0.83 (0.48 to 1.41)	0.482
IgM (0.5 g/L)	0.31 (0.21 to 0.47)	< 0.0001	0.43 (0.26 to 0.70)	0.001

Many determinants correlated with vaccine response on univariate analysis and five factors remained significant on multivariate assessment. Male gender was associated with a 44% reduction in the probability of developing an antibody response (*p* = 0.02) whilst this was 80% lower for patients on BTKi therapy (*p* < 0.0001). The presence of IgA or IgM hypogammaglobulinaemia and use of prophylactic antibiotic therapy for recurrent infection were also independent predictors of poor response with reductions of 72%, 57% and 72% in vaccine response, respectively (*p* > 0.0001; *p* = 0.001 and *p* = 0.027).

### Post vaccine sera from patients with CLL show poor neutralisation of the SARS-CoV-2 delta variant

Having determined the prevalence and magnitude of antibody responses to COVID-19 vaccination in patients with CLL we next went on to assess the relative functional activity of the antibody response. It was felt of particular importance to assess activity against the globally dominant delta SARS-CoV-2 variant and post-vaccine sera were therefore assessed for their ability to neutralise live SARS-CoV-2 infection in vitro using either the original prototypic Wuhan virus or the delta variant (lineage B.1.617.2).

Maximum percentage neutralisation was studied in post-vaccine sera from 94 participants and 94 age-matched controls. Median neutralisation of live Wuhan virus was 96% in healthy controls compared to 84% for the delta variant (*p* = 0.03).

**Fig. 3:**
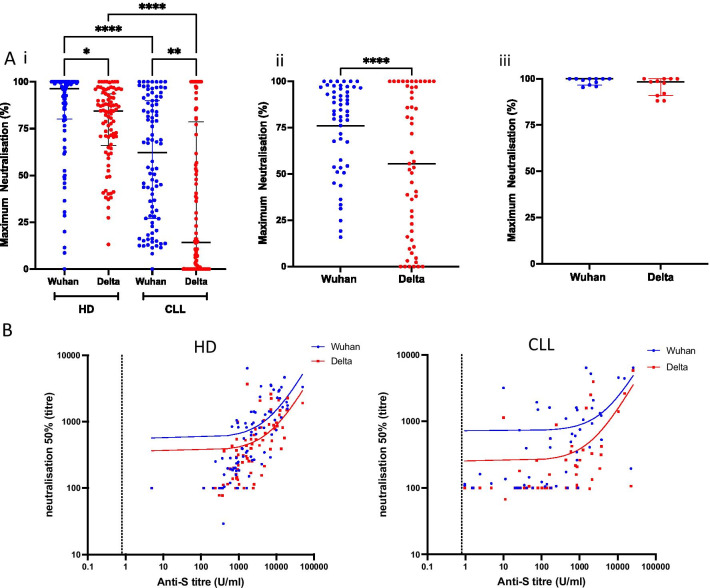
Post-vaccine neutralisation of live Wuhan virus and Delta variant. **A** (i) Maximum neutralisation of live virus using post-vaccine sera from healthy donors (HD; *n* = 94) and patients with CLL (*n* = 94) (*p* < 0.0001 Kruskal–Wallis). (ii) Maximum neutralisation in 53 CLL patients with a positive spike-specific antibody response following second vaccine (*p* < 0.0001). (iii) Maximum neutralisation using post-vaccine sera in HD and patients with serological evidence of previous natural infection with SARS-CoV-2. **B** Correlation between ND50 value against Wuhan virus or delta variant and spike-specific antibody titre in HD (Wuhan *r* = 0.80 and Delta *r* = 0.77; *p* < 0.0001) and CLL patients with a positive antibody response (Wuhan *r* = 0.60 and for Delta *r* = 0.61; *p* =  < 0.0001)

In contrast, maximal neutralisation of Wuhan virus was only 62% using sera from CLL patients (*p* < 0.0001 compared to healthy controls). Moreover, neutralisation of the delta variant compared to Wuhan virus was further reduced in CLL patients with a median value of only 14% (p = 0.007) (Fig. 3A (i)). The neutralisation capacity was next assessed in CLL participants with a positive spike-specific antibody response post-second vaccine (*n* = 53) and maximum neutralisation values of 84% and 56%, respectively, were observed against the Wuhan and Delta variant (*p* < 0.0001) (Fig. 3A (ii)). As such, post-vaccine sera from CLL patients show markedly reduced neutralisation capacity against the current pandemic delta variant.

Prior natural infection is known to increase the magnitude and functional quality of spike-specific antibody responses, and post-vaccine sera from both HD or CLL donors with previous natural infection exhibited equivalent neutralisation to Wuhan virus and delta variant with median values > 90% within both groups (Fig. 3A (iii)). Multivariable linear regression was then repeated using the same variables as listed in Table 2 to predict the maximum neutralisation percentage for both Wuhan and Delta variant amongst patients with CLL. For Wuhan virus, increasing age (− 0.12, 95% CI: − 2.04 to − 0.006; *p* = 0.049) and treatment for CLL (− 40, 95% CI: − 80 to − 0.78; *p* = 0.046) were both associated with impaired neutralisation. Male gender was the only independently predictive variable for neutralisation of delta variant, (− 21, 95% CI: − 43 to − 0.15; *p* = 0.049).

Next, serial dilutions were used to determine the reciprocal of the dilution that mediated 50% neutralisation (ND50) with the lowest dilution being 1/100. Median values against Wuhan virus were 399 and 479 for CLL and healthy donors, respectively, with markedly reduced values against the delta variant in the patient group (106 CLL vs 306 HD). ND50 values were then compared to Spike-specific antibody titre and were seen to correlate well, although accentuated loss of delta neutralisation was again seen in the patient group (Fig. 3B). Of interest, ND50 values were increased by 15–50-fold to reach 6400 in naturally infected donors.

This reduced ability to neutralise the delta variant in sera from the CLL patients was also apparent using a pseudotyped-virus neutralisation assay (Additional file 4: Figure S4).

## Discussion

Patients with CLL are at increased clinical risk following SARS-CoV-2 infection and it is essential that protective immune responses are optimised for this group. Here, we analyse results from one of the largest study of vaccine responses in patients with CLL and identify a range of novel findings to help guide patient management.

We found comparable rates of seroconversion to other studies (67%) but this compares poorly to values approaching 100% in age-matched control donors [6, 7, 17]. Furthermore, in those patients who do develop an antibody response, the median antibody titre is only 27% of that seen within age-matched controls. Nevertheless, the 294-fold increment observed between the first and second vaccines may augur well for the use of additional ‘booster’ vaccines. Previous natural infection with SARS-CoV-2 was seen in 2.8% of patients and leads to strong antibody responses, even following the first vaccination [5], and reveals strong immunological ‘priming’ after natural infection.

The relative immunogenicity of different COVID-19 vaccines has not been assessed previously in CLL patients. We found that the mRNA (BNT162b2) and adenovirus-based (ChAdOx1) vaccines elicited comparable antibody responses, and it will be important to assess this further with the additional vaccines in current global usage.

Vaccine response rates were highest in non-treated patients on expectant management, with 79% of patients seroconverting within the first 6 years of follow-up. This declined slightly to 76% amongst those 12 years or more since diagnosis and reflects the relative impairment in immune function that develops over time.

The lower response rates observed amongst patients about to commence treatment are likely to reflect the immunosuppressive nature of CLL disease burden and reinforce the need for appropriate therapeutic vaccine regimens to be delivered at diagnosis [18]. Antibody response rates were lowest in those on active treatment. Bruton Tyrosine Kinase inhibitor (BTKi) therapy is known to suppress vaccine responses as a reflection of its inhibition of B cell activation [19, 20], although we found response rates to be 17% higher in patients on first line therapy compared to treatment in relapse. Antibody response rates in patients on BCL-2 inhibitor therapy were also low at 29% although the cohort size was relatively modest. CD20-specific antibody therapy is used widely for patients with B cell malignancies and markedly impairs antibody responses to vaccine challenge. However, spike-specific responses improved by 38% in patients vaccinated more than 12 months since completion of therapy and suggest that vaccination should be encouraged in patients who are in remission at least one year after CD20 therapy.

In this large study, multivariate analysis identified five individual demographic and laboratory variables associated with a poor antibody response rates, providing additional risk factors to those previously identified [4, 5]. Male gender was associated with a 46% reduction in the probability of developing an antibody response. Higher vaccine-induced antibody responses in women have been reported previously [21] and are also seen in studies of COVID-19 vaccination [22]. Furthermore, low serum levels of IgA or IgM were independently associated with a 72% and 57% reduction in the probability of spike seroconversion after vaccination. This is not surprising given the high prevalence of hypogammaglobulinemia associated with CLL. Finally, in addition to current BTKi therapy, antibody response rates were reduced by 72% in patients taking prophylactic antibodies for recurrent infection. This risk factor has not previously been identified in other studies but provides an additional variable that is easy to identify within a clinical setting and likely reflects the clinical expression of immune impairment within this group [5, 6].

The longevity of antibody responses following COVID-19 vaccination to the differing vaccine platforms and extended interval is currently unknown and 3rd doses of vaccine are being deployed in many countries. We assessed antibody titres at 3–8 months after second vaccine and found these to fall by 33% during this period. This trajectory is broadly in line with previous reports from healthy donors [23] and is reassuring for patients who are able to generate an antibody response. This study will now continue to assess serological responses prospectively and following 3rd vaccination.

Current COVID-19 vaccines contain the spike protein from the original Wuhan SARS-CoV-2 virus. However, the delta variant is now ubiquitous and as such we assessed how post-vaccine serum samples compared in relation to relative neutralisation of the prototypic Wuhan virus and delta variant. Serum from age-matched control donors showed excellent neutralisation of Wuhan with only a modest decrease against live delta variant [24]. However, sera from patients with CLL who had seroconverted displayed a profound loss of neutralisation against live delta variant. This indicates impairment in both the magnitude and functional quality of the spike-specific antibody response in CLL patients. Poor functionality activity of antibody responses from CLL patients has been seen in other settings but these findings are of concern as they indicate the vulnerability of the CLL patient cohort to the globally dominant delta variant [13]. Indeed, recently published data from the EPICOVIDEHA registry, of which 25% of patients had a CLL diagnosis, continues to show mortality rates of around 12.5% amongst vaccinated patients [25].

The limitations of our study include relative low numbers of patients on venetoclax. Furthermore, no patients were on active immune-chemotherapy and as sero-conversion rates on this therapy are low it is possible that this may have acted to enhance the overall seroconversion rate within the cohort. Our studies used the Roche Elecsys platform for antibody quantification, a widely employed clinical assay, and it will be of interest to relate different assay systems to WHO serological standards in future studies. Cellular immunity is also important in protection against COVID-19 where it may be particularly effective against variants of concern [26]. As such, understanding how cellular immunity may contribute to clinical protection in patients with CLL is now paramount, particularly in patients receiving therapy which limits antibody generation.

## Conclusions

In conclusion, we report reduced spike-specific antibody responses, irrespective of the vaccine platform received, and show impaired neutralisation of the dominant delta variant after COVID-19 vaccination in patients with CLL. These findings argue strongly that further protection for this vulnerable cohort is needed. A third vaccine dose, homologous or heterologous to the initial immunogen [27], is likely to boost antibody responses in some patients [28]. However, hypogammaglobulinaemia is an important complication of CLL and patients with no measurable antibody response may require additional interventions including prophylactic antibody therapy.

## Supplementary Information

**Additional file 1.** A comparison of anti-spike titre measured by Roche using matched serum and eluate from dried blood spot samples. Legend: Strong positive correlation in anti-spike titre is shown between matched serum and eluate samples (r = 0.98; p < 0.0001).

**Additional file 2.** Comparison of antibody titre by vaccine interval. Legend: A comparison of anti-spike titre as measured by Roche in those who received the 2 doses on a standard interval compared to those donors who received it on an extended interval (p = 0.0014).

**Additional file 3.** Correlation of total serum Immunoglobulin compared to time since diagnosis amongst untreated patients. Legend: Correlation of serum immunoglobulin levels in untreated patients compared to time since diagnosis is shown; Spearman’s rank correlation: IgA (r = -0.019; p = 0.759) IgG (r = -0.07; p = 0.23) IgM (r = -0.15; p = 0.01).

**Additional file 4.** Pseudotype neutralization assay results at 50% neutralization. Legend: Antibody titres at 50% neutralization using pseudotype assay is shown. A reduction in neutralization titre was found in CLL patients for the delta variant compared to Wuhan prototype (p < 0.0001). This difference was less pronounced in healthy controls (p = 0.026). Inferior neutralization to the delta variant is shown in CLL patients compared to HD (p = 0.047).

## Abbreviations

HD: Healthy donors
IVIg: Immunoglobulin replacement therapy
CLL: Chronic Lymphocytic Leukaemia
CLL-VR: Chronic Lymphocytic Leukaemia—vaccine response
BTKi: Bruton tyrosine kinase inhibitor
CIA: Coronavirus immunological analysis
UPH: Urgent public health
IRAS: Integrated research application system
BCL2: B cell lymphoma 2

## Declarations

### Availability of data and materials

All datasets used during the current study are available from the corresponding author on reasonable request.
