# Supplementary material for: Impaired neutralisation of SARS-CoV-2 delta variant in vaccinated patients with B cell chronic lymphocytic leukaemia
Source: J Hematol Oncol. 2022 Jan 9;15:3. doi: 10.1186/s13045-021-01219-7 (PMC8743056; doi:10.1186/s13045-021-01219-7)
Supplement: Supplementary file 1 — Additional file 1. A comparison of anti-spike titre measured by Roche using matched serum and eluate from dried blood spot samples. Legend: Strong positive correlation in anti-spike titre is shown between matched serum and eluate samples (r = 0.98; p < 0.0001). [file 13045_2021_1219_MOESM1_ESM.pptx]

## Slide 1
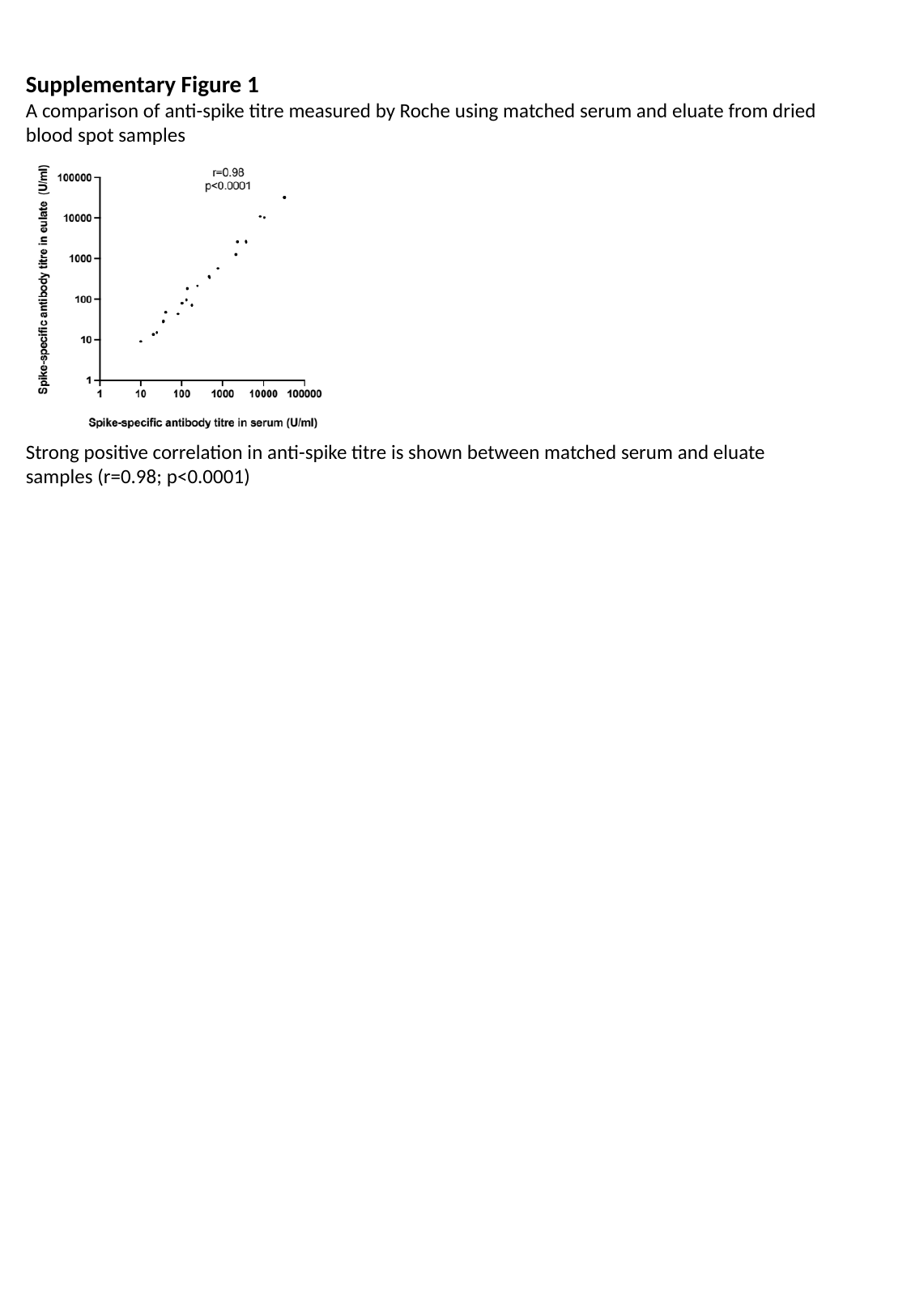

Supplementary Figure 1
A comparison of anti-spike titre measured by Roche using matched serum and eluate from dried blood spot samples
Strong positive correlation in anti-spike titre is shown between matched serum and eluate samples (r=0.98; p<0.0001)
