# Supplementary material for: Impaired neutralisation of SARS-CoV-2 delta variant in vaccinated patients with B cell chronic lymphocytic leukaemia
Source: J Hematol Oncol. 2022 Jan 9;15:3. doi: 10.1186/s13045-021-01219-7 (PMC8743056; doi:10.1186/s13045-021-01219-7)
Supplement: Supplementary file 2 — Additional file 2. Comparison of antibody titre by vaccine interval. Legend: A comparison of anti-spike titre as measured by Roche in those who received the 2 doses on a standard interval compared to those donors who received it on an extended interval (p = 0.0014). [file 13045_2021_1219_MOESM2_ESM.pptx]

## Slide 1
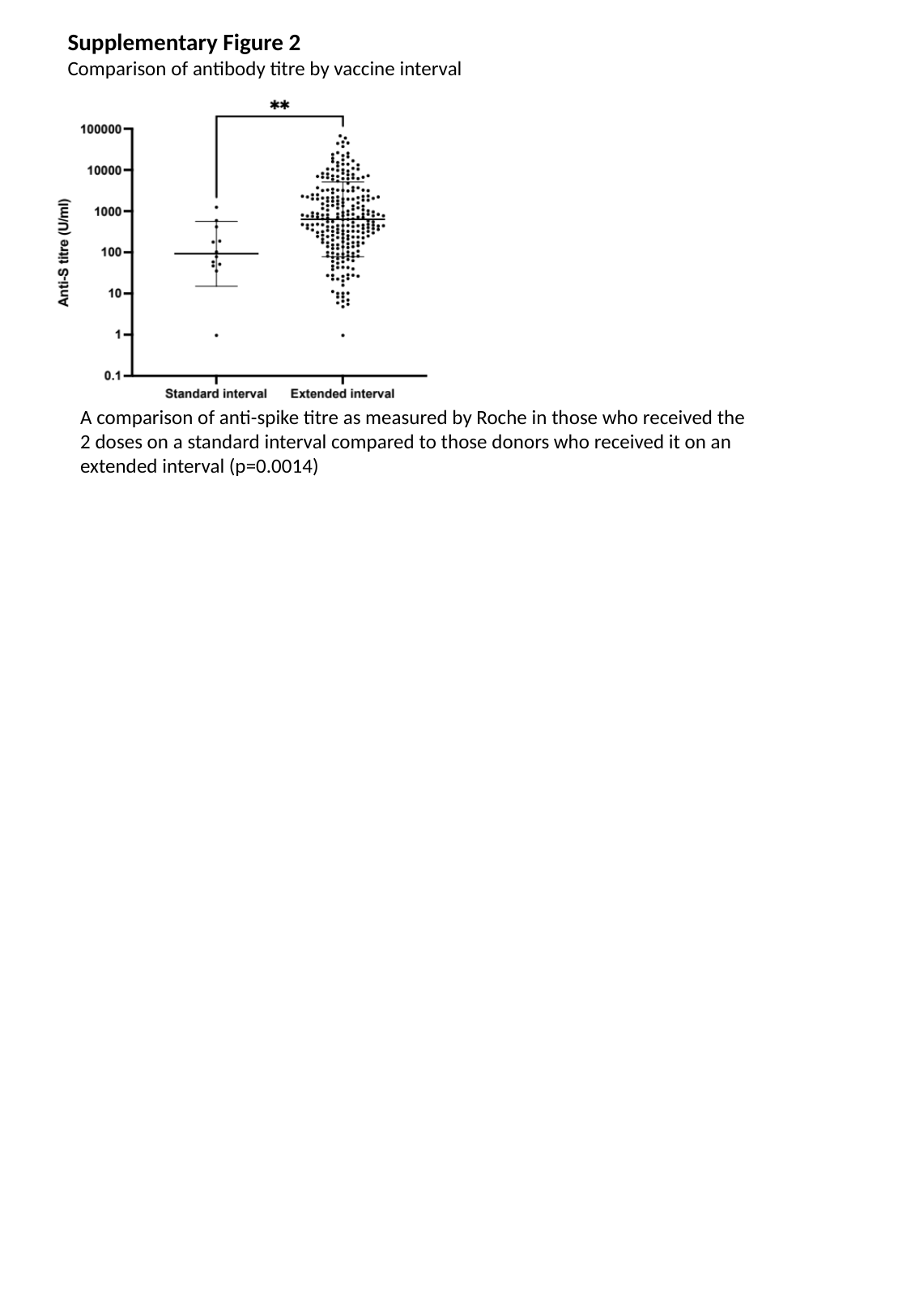

Supplementary Figure 2
Comparison of antibody titre by vaccine interval
A comparison of anti-spike titre as measured by Roche in those who received the 2 doses on a standard interval compared to those donors who received it on an extended interval (p=0.0014)
