# Supplementary material for: Impaired neutralisation of SARS-CoV-2 delta variant in vaccinated patients with B cell chronic lymphocytic leukaemia
Source: J Hematol Oncol. 2022 Jan 9;15:3. doi: 10.1186/s13045-021-01219-7 (PMC8743056; doi:10.1186/s13045-021-01219-7)
Supplement: Supplementary file 3 — Additional file 3. Correlation of total serum Immunoglobulin compared to time since diagnosis amongst untreated patients. Legend: Correlation of serum immunoglobulin levels in untreated patients compared to time since diagnosis is shown; Spearman’s rank correlation: IgA (r = -0.019; p = 0.759) IgG (r = -0.07; p = 0.23) IgM (r = -0.15; p = 0.01). [file 13045_2021_1219_MOESM3_ESM.pptx]

## Slide 1
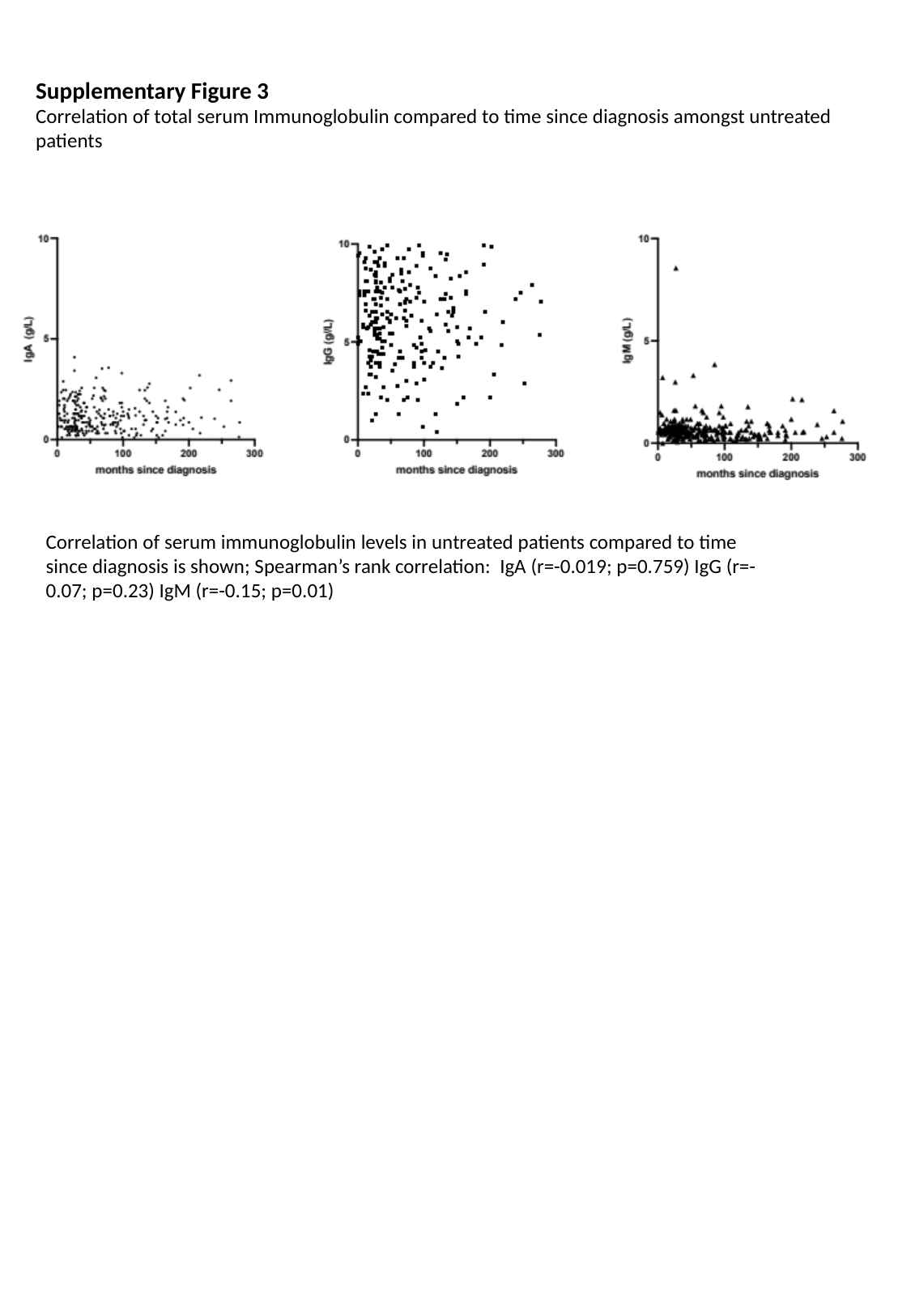

Supplementary Figure 3
Correlation of total serum Immunoglobulin compared to time since diagnosis amongst untreated patients
Correlation of serum immunoglobulin levels in untreated patients compared to time since diagnosis is shown; Spearman’s rank correlation: IgA (r=-0.019; p=0.759) IgG (r=-0.07; p=0.23) IgM (r=-0.15; p=0.01)
