# Supplementary material for: Impaired neutralisation of SARS-CoV-2 delta variant in vaccinated patients with B cell chronic lymphocytic leukaemia
Source: J Hematol Oncol. 2022 Jan 9;15:3. doi: 10.1186/s13045-021-01219-7 (PMC8743056; doi:10.1186/s13045-021-01219-7)
Supplement: Supplementary file 4 — Additional file 4. Pseudotype neutralization assay results at 50% neutralization. Legend: Antibody titres at 50% neutralization using pseudotype assay is shown. A reduction in neutralization titre was found in CLL patients for the delta variant compared to Wuhan prototype (p < 0.0001). This difference was less pronounced in healthy controls (p = 0.026). Inferior neutralization to the delta variant is shown in CLL patients compared to HD (p = 0.047). [file 13045_2021_1219_MOESM4_ESM.pptx]

## Slide 1
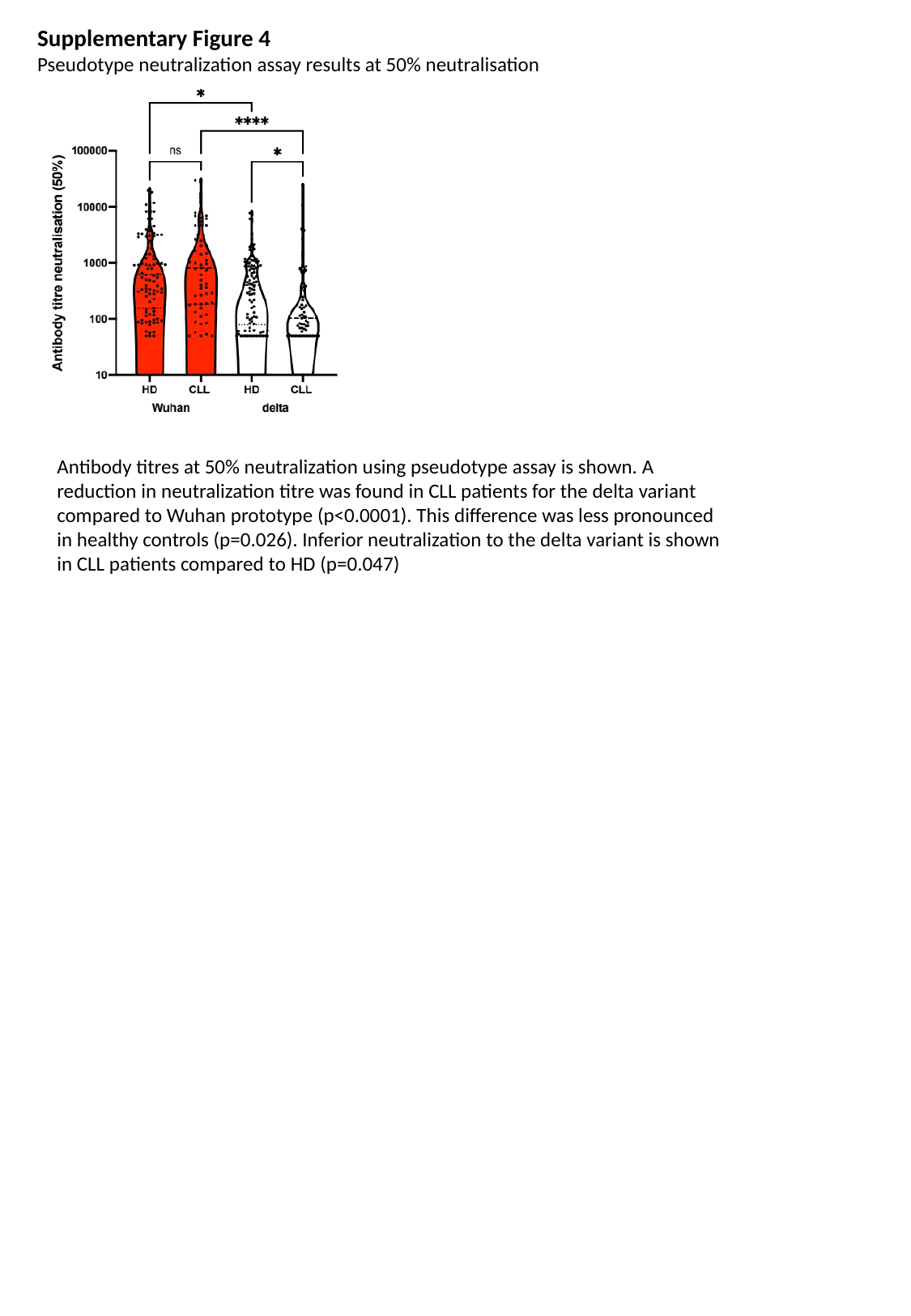

Supplementary Figure 4
Pseudotype neutralization assay results at 50% neutralisation
#
Antibody titres at 50% neutralization using pseudotype assay is shown. A reduction in neutralization titre was found in CLL patients for the delta variant compared to Wuhan prototype (p<0.0001). This difference was less pronounced in healthy controls (p=0.026). Inferior neutralization to the delta variant is shown in CLL patients compared to HD (p=0.047)
